# RETRACTION: Effect of Tobacco Usage on Surgical Site Wound Problems After Primary Total Hip and Total Knee Arthroplasty: A Meta‐Analysis

**DOI:** 10.1111/iwj.70261

**Published:** 2025-02-25

## Abstract

**RETRACTION:** ZhuJ. , SiM. , and HuangZ. , “Effect of Tobacco Usage on Surgical Site Wound Problems After Primary Total Hip and Total Knee Arthroplasty: A Meta‐Analysis,” International Wound Journal 21, no. 1 (2024): e14375, 10.1111/iwj.14375.37675771 PMC10784423

The above article, published online on 07 September 2023, in Wiley Online Library (http://onlinelibrary.wiley.com/), has been retracted by agreement between the journal Editor in Chief, Professor Keith Harding; and John Wiley & Sons Ltd. Following an investigation by the publisher, all parties have concluded that this article was accepted solely on the basis of a compromised peer review process. The editors have therefore decided to retract the article. The authors did not respond to our notice regarding the retraction.

**RETRACTION:** J. Zhu , M. Si , and Z. Huang , “Effect of Tobacco Usage on Surgical Site Wound Problems After Primary Total Hip and Total Knee Arthroplasty: A Meta‐Analysis,” International Wound Journal 21, no. 1 (2024): e14375, 10.1111/iwj.14375.37675771 PMC10784423

The above article, published online on 07 September 2023, in Wiley Online Library (http://onlinelibrary.wiley.com/), has been retracted by agreement between the journal Editor in Chief, Professor Keith Harding; and John Wiley & Sons Ltd. Following an investigation by the publisher, all parties have concluded that this article was accepted solely on the basis of a compromised peer review process. The editors have therefore decided to retract the article. The authors did not respond to our notice regarding the retraction.

